# In vitro and in vivo antitrypanosomal activity of the fresh leaves of *Ranunculus Multifidus* Forsk and its major compound anemonin against *Trypanosoma congolense* field isolate

**DOI:** 10.1186/s12917-023-03856-1

**Published:** 2024-01-27

**Authors:** Betelhem Sirak, Gizachew Kassahun Bizuneh, Peter Imming, Kaleab Asres

**Affiliations:** 1 Department of Pharmacy, College of Medicine and Health Sciences, Arba Minch University, P.O. Box 21, Arba Minch, Ethiopia; 2 Department of Pharmacognosy, School of Pharmacy, College of Medicine and Health Sciences, University of Gondar, P.O. Box 196, Gondar, Ethiopia; 3 Institute of Pharmacy, Martin Luther University Halle-Wittenberg, Kurt-Mothes-Str. 3, 06120 Halle (Saale), Germany; 4 Department of Pharmaceutical Chemistry and Pharmacognosy, School of Pharmacy, College of Health Sciences, Addis Ababa University, P.O. Box 1176, Addis Ababa, Ethiopia

**Keywords:** *Ranunculus multifidus*, Fresh leaves, Antitrypanosomal activity, *Trypanosoma congolense*, Anemonin

## Abstract

**Background:**

Animal trypanosomiasis is a major livestock problem due to its socioeconomic impacts in tropical countries. Currently used trypanocides are toxic, expensive, and the parasites have developed resistance to the existing drugs, which calls for an urgent need of new effective and safe chemotherapeutic agents from alternative sources such as medicinal plants. In Ethiopian traditional medicine fresh leaves of *Ranunculus multifidus* Forsk, are used for the treatment of animal trypanosomiasis. The present study aimed to evaluate the antitrypanosomal activity of the fresh leaves of *R. multifidus* and its major compound anemonin against *Trypanosoma congolense* field isolate.

**Methods:**

Fresh leaves of *R. multifidus* were extracted by maceration with 80% methanol and hydro-distillation to obtain the corresponding extracts. Anemonin was isolated from the hydro-distilled extract by preparative TLC. For the in vitro assay, 0.1, 0.4, 2 and 4 mg/ml of the test substances were incubated with parasites and cessation or drop in motility of the parasites was monitored for a total duration of 1 h. In the in vivo assay, the test substances were administered intraperitoneally daily for 7 days to mice infected with *Trypanosoma congolense*. Diminazene aceturate and 1% dimethylsulfoxide (DMSO) were used as positive and negative controls, respectively.

**Results:**

Both extracts showed antitrypanosomal activity although the hydro-distilled extract demonstrated superior activity compared to the hydroalcoholic extract. At a concentration of 4 mg/ml, the hydro-distilled extract drastically reduced motility of trypanosomes within 20 min. Similarly, anemonin at the same concentration completely immobilized trypanosomes within 5 min of incubation, while diminazene aceturate (28.00 mg/kg/day) immobilized the parasites within 10 min. In the in vivo antitrypanosomal assay, anemonin eliminates parasites at all the tested doses (8.75, 17.00 and 35.00 mg/kg/day) and prevented relapse, while in diminazene aceturate-treated mice the parasites reappeared on days 12 to 14.

**Conclusions:**

The current study demonstrated that the fresh leaves of *R. multifidus* possess genuine antitrypanosomal activity supporting the use of the plant for the treatment of animal trypanosomiasis in traditional medicine. Furthermore, anemonin appears to be responsible for the activity suggesting its potential as a scaffold for the development of safe and cost effective antitrypanosomal agent.

**Supplementary Information:**

The online version contains supplementary material available at 10.1186/s12917-023-03856-1.

## Background

Trypanosomiasis is an infectious disease caused by parasitic protozoan trypanosomes of the genus *Trypanosoma*. It is a major livestock problem due to its socioeconomic impact such as mortality, morbidity, reduction in milk and meat production, abortion, and costs associated with treatment [1, 2]. Animal trypanosomiasis is prevalent in the southern and western regions of Ethiopia where the primary vector tsetse-fly (*Glossina pallidipes*) exists [3]. In Ethiopia, bovine trypanosomiasis is caused by *Trypanosoma congolense*, the dominant trypanosome species, followed by *T. vivax* and *T. brucei brucei* [1, 4]. Approaches to control trypanosomiasis include control or elimination of tsetse flies by control of breeding sites of vector and use of insecticides and sterile male techniques [4, 5]. The other is prevention or treatment of animals using trypanocidal drugs. These approaches, however, have many drawbacks such as high cost of drugs and insecticides, possibilities of undesirable environmental pollution by insecticides, and increasing development of resistance in the parasites to the existing drugs and toxicity [6]. The development of drug resistance and absence of vaccine against trypanosomes calls for the urgent need of new drugs for the control of trypanosomiasis from alternative sources such as medicinal plants [6, 7].

The genus *Ranunculus* belongs to the family *Ranunculaceae* commonly known as a buttercup family [8]. In Ethiopia, the genus is represented by 10 species of which *Ranunculus multifidus* Forsk. is indigenous to the country [9]. In Gamo zone, south-west Ethiopia, fresh leaves of *R. multifidus* are chopped, squeezed and filtered to prepare the juice which is given to cattle by oral drenching to treat oedema and trypanosomiasis [10]. Similarly, in Bensa Woreda, Southern Ethiopia, crushed leaf mixed with water is used to treat livestock mastitis, internalparasite and trypanosomiasis [11]. Despite the well documented antitrypanosomal claims of *R. multifidus*, to date, there appears to have been no report in the literature concerning the antitrypanosomal activity of the plant. Hence, this study was conducted to evaluate the possible antitrypanosomal activities of the fresh leaves of *R. multifidus* and its major constituent, anemonin. It is believed that such endeavors give impetus to developing countries as they might provide an opportunity to exploit their natural flora for resolving autochthonous health problems. They also offer the potential to discover secondary metabolites that can directly be used as drug or serve as lead molecules for drug discovery.

## Materials and methods

### Plant material

Fresh leaves of *R. multifidus* were collected from Dorze village of Chencha woreda, Gamo zone, (520 km southwest of Addis Ababa, Ethiopia) located in the Rift Valley above the west shore of Lake Abaya at 6°11′36″ N and 37°34′13″ E in August, 2019. The plant material was authenticated by Ato Melaku Wondafrash, National Herbarium, Department of Biology, College of Natural and Computational Sciences, Addis Ababa University (AAU), where a botanical specimen was deposited (collection number BS-001) for future reference.

### Chemicals and reference drug

The chemicals and reagents used for the experiment include methanol (Cheshire, UK), dimethyl sulfoxide (DMSO) (Merck KGaA, Darmstadt, Germany), phosphate buffered saline (PBS) (Shenyang Xin Guang, China), and diminazene diaceturate BP Vet [4,4- (diazoamino) dibenzamidine diaceturate] + 1.31 g phenazone BP (Ashish, India). All compounds prepared were characterised by ^1^H, ^13^C NMR and ESI–MS spectra and found to correspond to the expected constitution.

### Experimental animals and parasite

Swiss albino mice of either sex weighing 22–30 g and age 5–6 weeks were employed throughout the experiment. The mice were obtained from the animal house of the Department of Pharmacology (DoP), School of Pharmacy (SoP), College of Health Sciences (CHS), AAU. The animals were held in stainless steel cages at room temperature and a 12 h light/12 h dark cycle. They were provided with water and food pellets ad libitum in the animal house of the DoP, SoP, CHS, AAU. All the experiments were conducted in accordance with the internationally accepted laboratory animal use and care guideline [12] and were approved by the Institutional Review Board of the SoP, AAU (approval code: ERB/SOP241b/13/2021). *Trypanosoma congolense* was obtained from naturally infected cattle in Abulo Kebele, Arba Minch, Gamo zone, South Ethiopia. The parasites were maintained in the laboratory by serial blood passage from infected mice to the non-infected ones.

### Extraction and isolation

Fresh leaves of *R. multifidus* (2 kg) were crushed and macerated with 6 L of 80% methanol at room temperature for 72 h immediately after collection. This was repeated twice and the combined filtrate was concentrated in a rotavapor (Buchi, Flawil, Switzerland) at a temperature not exceeding 40 °C. The remaining aqueous solution was dried in a lyophilizer. The dried extract labeled as RM-M (80% methanol extract of *Ranunculus multifidus*) was transferred to an amber-coloured bottle and stored in a refrigerator at 4 °C until use.

Fresh leaves of *R. multifidus* (1 kg) were chopped into small pieces and subjected to hydrodistillation for 3 h using a Clevenger type apparatus. The condensate was collected and extracted with 100 ml chloroform (3 ×) using a separatory funnel. To the combined organic solvent extract, about 5 g of ahydrous sodium sulfate was added to remove moisture and then filtered using Whatman no 1 filter paper. The organic solvent was concentrated in a rotavapor at a temperature not exceeding 35 °C to yield a pungent oil designated RM-H (hydro-distilled extract of *Ranunculus multifidus*). RM-H was transferred into an amber-coloured vial and stored in a refrigerator at 4 °C for further experiment.

RM-H was subjected to preparative thin layer chromatography (PTLC) and the chromatograms were developed using a mixture of *n*-hexane and ethyl acetate (5:1) as a mobile phase and visualized using ultraviolet light (UV) of wave lengths 254 nm and 366 nm (CAMAG, Muttenz, Switzerland). The major band was carefully scrapped off from the plates, washed with a mixture of chloroform and methanol (1:1), filtered using Whatman no. 1 filter paper and concentrated to dryness under reduced pressure. The white powder obtained was further purified by PTLC using ethyl acetate: hexane (3:2) as a solvent system, weighed, transferred into an amber-coloured vial and stored in a refrigerator at ˗4 °C until use. The compound was characterized as anemonin on the basis of FT-IR, APCI-MS, 1D- and 2D-NMR spectral assignments and also by comparison with the reported spectroscopic data described previously [13].

### Preparation of Fmoc-protected glutathione methylester

Fmoc-protected glutathione methylester was prepared by the following sequence of reactions described in the literature [14–16].

### Synthesis of N-[(9H-fluoren-9-ylmethoxy)carbonyl]-L-γ-glutamyl-L-cysteinyl-glycine, bimol. (2 → 2′)-disulfide

N-[(9H-fluoren-9-ylmethoxy)carbonyl]-L-γ-glutamyl-L-cysteinyl-glycine, bimol. (2 → 2′)-disulfide was synthesized by the method described by Narita et al. [14]. A solution of 9-fluorenylmethyl chloroformate (FmocCl) in dioxane (6.5 ml) was added dropwise to a stirred solution of commercial glutathione disulfide in 10% aqueous Na_2_CO_3_ (6.5 ml) at room temperature. After 2 h, the reaction mixture was diluted with H_2_O (85 ml) and the resulting mixture was washed with diethyl ether (2 × 40 ml). Concentrated H_2_SO_4_ was slowly added to the aqueous layer until the pH was 2–3 and the resulting solution was extracted with EtOAc (3 × 100 ml). The combined extracts were washed with H_2_O (2 × 50 ml) and brine (2 × 50 ml) and then dried over Na_2_SO_4_. Concentration of the solvent in vacuo gave 0.7 g (54%) of the title compound.

### Synthesis of N-[(9H-fluoren-9-ylmethoxy)carbonyl]-L-γ-glutamyl-L-cysteinyl-glycine dimethylester, bimol. (2 → 2′)-disulfide

N-[(9H-fluoren-9-ylmethoxy)carbonyl]-L-γ-glutamyl-L-cysteinyl-glycine dimethylester, bimol. (2 → 2′)-disulfide was dissolved in methanol and cooled in an ice bath. Oxalyl chloride (6 eq., 95 μl) was added slowly and the reaction vessel tightly closed. After 4 h, another batch of oxalyl chloride (6 equivalents, 95 μl) was added. The reaction was stirred for an additional 8 h. The solvent was removed under reduced pressure [15]. The residue (66 mg, 31%, white solid) was used for the next step without further purification.

### Synthesis of N-[(9H-fluoren-9-ylmethoxy)carbonyl]-L-γ-glutamyl-L-cysteinyl-glycine dimethylester

All steps were performed under an Argon atmosphere. To an ice-cold solution of N-[(9H-fluoren-9-ylmethoxy)carbonyl]-L-γ-glutamyl-L-cysteinyl-glycine dichloride, bimol. (2 → 2′)-disulfide in THF (2.50 ml), a solution of NaBH_4_ in MeOH (2 ml) was added dropwise. The resulting mixture is stirred at room temperature for 10 min, cooled to 0 °C and the reaction quenched by adding a saturated aqueous NH_4_Cl solution (3 ml). The mixture was diluted with EtOAc (20 ml), the phases are separated and the aqueous phase extracted with EtOAc (3 × 20 ml). The combined organic extracts were washed with brine, dried over anhydrous MgSO_4_ and the solvent was removed under reduced pressure to obtain the product (43 mg, 66%, white solid) [16].

### Incubation with anemonin

All steps were performed under an Argon atmosphere. The protected glutathione derivative (N-[(9H-fluoren-9-ylmethoxy)carbonyl]-L-γ-glutamyl-L-cysteinyl-glycine dimethylester; 10 mg) was tested for purity by TLC, particularly in view of the facile reoxidation to the corresponding disulfide, and dissolved in DMSO-d_6_ (1 ml). Anemonin (3 mg; isolated by us from *R. multifidus*) was added, and ^1^H NMR spectra were measured after 15 min, 2 h, 4 h, 1 day and 6 days. Since the spectrum remained unchanged, the solution was heated to 60 °C for 1 h, still leading to no change in the ^1^H NMR spectrum and on TLC.

### Acute oral toxicity

Acute oral toxicity study was carried out as per the internationally accepted protocol of OECD Guideline 425 [17] using limit and main testes, as described earlier [13].

### Antitrypanosomal assay

#### Determination of parasitemia

Parasitemia was monitored in blood obtained from the tail of mice. The number of parasites was counted by microscope at 40 × magnification using the “Rapid Matching” method, which involves microscopic counting of parasites per field in infected blood and logarithm values of trypanosome number were obtained by matching with the table converted to Log number to provide absolute number of trypanosomes per ml of blood [18].

### In vitro assay

In 96-well plates, 200 μl of blood containing about 20*–*25 parasites per field were mixed with 50 μl of the test substances at concentrations of 0.5, 2, 10 and 20 mg/ml to produce effective test concentrations of 0.1, 0.4, 2 and 4 mg/ml, respectively. The dose was determined from previous in vitro studies [19–21]*.* Parasites suspended in 50 μl of 1% DMSO were used as a negative control. Similar doses of diminazene aceturate were used as a positive control [20–22]*.* The plates were incubated at 37 °C for 5 min, then about 2 μl of the test mixtures were placed on separate microscope slides and covered with a coverslip (22 × 22 mm) and the parasites were observed every 5 min for a total duration of 1 h [23–25]*.* Cessation or drop in motility of the parasites in test sample-treated blood compared to the negative control was taken as a measure of trypanocidal activity [22, 26, 27]*. *In vitro trypanocidal activity was performed in triplicate experiments with each test concentration done in duplicate.

#### In vivo infectivity test

Infectivity test was performed in order to know if there are any remaining infective parasites after in vitro tests [28]. The remaining incubation mixtures (0.2 ml) from the in vitro assay were inoculated into healthy mice (5 mice per dose) intraperitoneally then the mice were monitored for development of parasitemia for 30 days [20, 21].

### In vivo assay

Fifty-five mice were injected intraperitoneally with 0.2 ml of *T. congolense* infected blood diluted with PBS containing approximately 10^5^ trypanosome cells collected from donor mouse by cardiac puncture [29]. The mice were left to develop parasitemia for 2 weeks until the average parasitemia became approximately 7.20 (Log number) ~ 10^7.20^/ml [26]. After 2 weeks, the mice were randomly divided into eleven groups with five mice in each group. Treatment started on the 14th day post-infection (day 0 of treatment). Mice in Group 1 injected with vehicle (1% DMSO, 10 ml/kg/day) served as a negative control, while mice in Group 2 injected with a single dose of diminazene aceturate (28.00 mg/kg) served as a positive control. The remaining nine groups served as treatment groups. Animals in Groups 3—5 were injected with 100, 200 and 400 mg/kg of RM-M, respectively, while animals in Groups 6—8 and Groups 9—11 were injected 8.75, 17.50 and 35.00 mg/kg of RM-H and anemonin, respectively. Dose selection for each group was based on oral acute toxicity results in which the middle dose was one tenth of the safe dose (~ 2000 mg/kg for RM-M and ~ 175 mg/kg for RM-H and anemonin). The higher dose was twice the middle dose, and the lower dose was half of the middle dose [30, 31]. All test substances were dissolved in 1% DMSO and administered intraperitoneally every morning for seven days with continuous monitoring of parasitemia every other day until the end of the experiment at the 14th day [30]. For the assessment of antitrypanosomal effect of the test substances, the level of parasitemia was expressed as Log number of parasites per ml of blood [32, 33]. A thin smear was made from a drop of blood obtained from the tail of a mouse on microscope slides and monitored for parasitemia every other day at 400 × total magnification. Parasitemia level was determined using the “Rapid Matching” technique of Herbert and Lumsden [18]. Wet blood smear was prepared in triplicate and the slide counts mean value was taken per sample. Body weight, rectal temperature and PCV were measured just before infecting the mice (pre-infection), just before starting treatment (day 0), at the end of the treatment (day 7) and at end of the experiment (day 14). Five healthy uninfected and untreated mice were used for comparison of the three parameters (body weight, rectal temperature and PCV) [22, 30]. The animals were euthanized by cervical dislocation after completion of the experiment.

### Determination of packed cell volume (PCV), rectal temperature and body weight

Heparinized microhematocrit capillary tubes were used to collect blood from tail of each mouse, 3/4th of the tube and sealed at their dry end with sealing clay [34]. The tubes were then placed in a microhematocrit centrifuge with the sealed ends outwards [35]. The blood was centrifuged at 12,000 rpm for 10 min [36]. PCV was determined using the following relation.

$$\text{PCV}=\frac{\mathrm{Volume}\;\mathrm{of}\;\mathrm{erythrocytes}\;\mathrm{in\;a}\;\mathrm{given}\;\mathrm{volume}\;\mathrm{of}\;\mathrm{blood}}{\mathrm{Total}\;\mathrm{blood}\;\mathrm{volume}}\times100$$

All mice were weighed using a sensitive digital weighing balance, and digital rectal thermometer was used to measure rectal temperature.

### Statistical analysis

Data analysis was carried out using IBM SPSS (Statistical Package for Social Sciences) Statistics for Windows, Version 25.0. Results were expressed as mean ± standard error of mean (mean ± SEM). Statistical significance was determined by one-way ANOVA followed by Tukey post hoc test to compare different parameters among the treatment and control groups. *p* < 0.05 was considered significant.

## Results and discussion

### In vitro activity

**Fig. 1:**
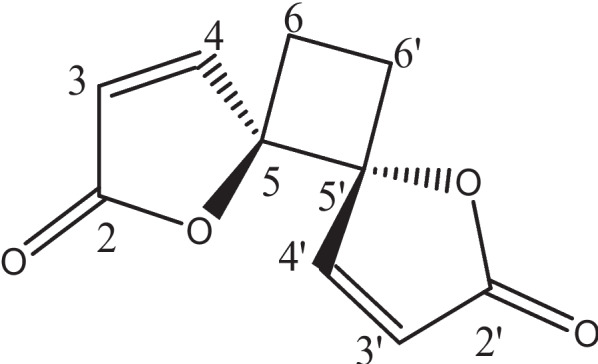
Structural formula of anemonin

In view of the traditional use of *R. multifidus* for the treatment of animal trypanosomiasis, it was of interest to evaluate the antitrypanosomal activity of crude extracts and anemonin (Fig. 1) isolated from fresh leaves of the plant. In vitro and in vivo antitrypanosomal activities were assessed against *T. congolense* field isolate, which is the dominant bovine trypanosome species causing African animal trypanosomiasis in Ethiopia.

In the current study, preliminary antitrypanosomal activity screening was conducted by an in vitro method as it is relatively simple, cheap and reliable [37, 38]. The assay is based on parasite motility, which is considered to be a relatively reliable indicator of viability of most trypanosomes [39]. A complete elimination or reduction in motility of trypanosomes when compared to the control was taken as index of trypanocidal activity [26]. As shown in Table 1, at a concentration of 4 mg/ml, the hydro-distilled extract (RM-H) ceased motility of the trypanosomes within 20 min and drastically reduced motility within 25 and 45 min at concentrations of 2 and 0.4 mg/ml, respectively. Similarly, the 80% methanol extract (RM-M) reduced motility of the trypanosomes within 30, 45 and 55 min at 4, 2 and 0.4 mg/ml test concentrations, respectively. The negative control (1% DMSO) and the lower test concentration (0.1 mg/ml) of both RM-H and RM-M did not immobilize or reduce motility of trypanosomes. Furthermore, at 4 mg/ml test concentration, anemonin ceased motility of trypanosomes within 5 min and drastically reduced motility within 15, 20 and 35 min at 2, 0.4 and 0.1 mg/ml test concentrations, respectively, whereas the positive control diminazene aceturate immobilized motility of trypanosomes within 10, 15, 30 and 45 min at 4, 2, 0.4 and 0.1 mg/ml test concentrations, respectively (Table 1).

**Table 1:** In vitro antitrypanosomal effect of the leaf extracts of *Ranunculus multifidus* and anemonin on *Trypanosoma congolense* motility and in vivo infectivity test

Dose (mg/ml)	Effect	1% DMSO	DA	RM-M	RM-H	Anemonin
4.0	Change in motility (in min)	NE	10^a^	30^b^	20^a^	5^a^
	No of infectious mice / Total no of mice	5/5	0/5	2/5	0/5	0/5
	Infection interval (in days)	12 ± 0.00	Ni	18 ± 0.34	Ni	Ni
2.0	Change in motility (in min)	NE	15^b^	45^b^	25^b^	15^b^
	No of infectious mice / Total no of mice	5/5	1/5	3/5	2/5	1/5
	Infection interval (in days)	12 ± 0.00	22	16 ± 0.48	18 ± 0.50	22
0.4	Change in motility (in min)	NE	30^b^	50^b^	45^b^	20^b^
	No of infectious mice / Total no of mice	5/5	3/5	5/5	4/5	2/5
	Infection interval (in days)	12 ± 0.00	16 ± 0.56	12 ± 0.00	14 ± 0.37	18 ± 0.00
0.1	Change in motility (in min)	NE	45^b^	NE	NE	35^b^
	No of infectious mice / Total no of mice	5/5	4/5	5/5	5/5	3/5
	Infection interval (in days)	12 ± 0.00	14 ± 0.48	12 ± 0.00	12 ± 0.00	16 ± 0.37

Data are expressed as mean ± SEM; *n* = 5; Ni: no infection; DA: diminazene aceturate; 1% dimethyl sulfoxide (DMSO): vehicle; RM-M: 80% methanol extract of *Ranunculus multifidus*, RM-H: hydro-distilled extract of *Ranunculus multifidus*; ^a^motility ceased; ^b^motility drastically reduced; *NE* No effect on motility. The experiments were performed in triplicate with each test concentration done in duplicate. The numbers under change in motility means the time (in minute) in which the change of motility is observed

In the in vivo infectivity test, all infected mice which received 4 mg/ml of RM-H, anemonin and diminazene aceturate were found to be free from parasite (lost infectivity) during the study period of 30 days. All the animals in the negative control group, RM-M (0.1 and 0.4 mg/ml) and RM-H (0.1 mg/ml) treated groups developed infection on day 12 (Table 1).

Results of the present study showed that RM-H possesses appreciable in vitro antitrypanosomal activity when compared to RM-M. Therefore, RM-H was subjected to phytochemical analysis which resulted in the isolation of a dilactone identified as anemonin. As shown in Table 1, the capacity of anemonin to cause trypanosomes lose their infectivity to mice was superior to the effects exerted by the crude extracts. However, in vitro antitrypanosomal activity displayed by the lower dose levels of anemonin could not be corroborated by blood incubation infectivity test suggesting that complete immobility of the parasites in vitro may not necessarily indicate that the parasites were dead. This result is consistent with a previous report by Yusuf et al. [19]. In that event, the lower dose levels might have only immobilized the parasite by causing unfavorable conditions, but not killed the parasites. As a consequence, when suitable physiological conditions are created the parasites might have recovered and become infective [31].

At a concentration of 4 mg/ml anemonin immobilized motility of trypanosomes within 5 min, while the standard dug diminazene aceturate at similar dose immobilized the parasites within 10 min. Therefore, anemonin can be considered a promising antitrypanosomal agent, since its activity was stronger and more rapid than that of the positive control.

### In vivo activity

Results of the present study demonstrated that mice treated with all dose levels of RM-H and RM-M showed low (*p* < 0.001) parasitemia level, throughout the observation period as compared to the negative control group. However, neither of the extracts completely eliminated the parasites from the infected mice blood stream, but only reduced (*p* < 0.001) the level of parasitemia as compared to the negative control. At a dose of 35.00 mg/kg, RM-H exhibited statistically significant (*p* < 0.001) parasitemia reduction compared to RM-M, lower (8.75 mg/kg) and middle doses (17.50 mg/kg) of RM-H. Mice treated with all dose levels of anemonin had significantly (*p* < 0.001) lowered parasitemia throughout the observation period compared to the negative control group. There was no statistical difference between the activity of diminazene aceturate and anemonin (Table 2). At all dose levels, anemonin eliminated trypanosomes from infected mice. It is interesting to note that mice treated with anemonin did not show relapse, while mice treated with diminazene aceturate showed no parasite growth from day 2 to 10, but relapse occurred in all mice on day 12–14 post-treatment initiation (Table 2). Previously, Tewabe et al. [20], Mergia et al. [30], and Ibrahim et al. [40] reported the occurrence of relapse in mice treated with diminazene aceturate. The relapse seen in mice treated with diminazene aceturate might be due to the emergence and spread of trypanocidal drug resistance in several African countries, including Ethiopia [41–43].

**Table 2:** In vivo antitrypanosomal effect of the leaf extracts of *Ranunculus multifidus* and anemonin on parasitemia level (Log number trypanosomes/ml) of *Trypanosoma congolense* infected mice

Test substance (mg/kg)	Log number trypanosomes/ml							
	**Day 0**	**Day 2**	**Day 4**	**Day 6**	**Day 8**	**Day 10**	**Day 12**	**Day 14**
1% DMSO	7.26 ± 0.06	7.74 ± 0.14	8.28 ± 0.07	8.58 ± 0.07	8.64 ± 0.06	8.70 ± 0.0	8.82 ± 0.07	8.94 ± 0.06
DA 28.00	7.32 ± 0.07	2.16 ± 1.32^a3^	0.00 ± 0.00^a3^	0.00 ± 0.00^a3^	0.00 ± 0.00^a3^	0.00 ± 0.00^a3^	1.08 ± 1.08^a3^	2.16 ± 1.32^a3^
RM-M 100	7.26 ± 0.06	6.90 ± 0.09^b3,(i−k)3^	6.6 ± 0.00^a3,b3,(h–k)3^	6.18 ± 0.20^a3,b3,(h–k)3^	4.86 ± 1.23^b1,(i−k)1^	6.30 ± 0.09^a3,b3,(h–k)3^	6.48 ± 0.07^a3, b3,(i−k)3^	6.78 ± 0.07^a3,b3,(i−k)3^
RM-M 200	7.32 ± 0.07	6.78 ± 0.12^b3,(i−k)3^	6.48 ± 0.07^a3,b3,(h–k)3^	5.88 ± 0.20^a3,b3,(h–k)3^	4.44 ± 1.11	6.12 ± 0.2^a3,b3,(h–k)3^	6.42 ± 0.07^a3,b3,(i−k)3^	6.60 ± 0.00^a3,b3,(i−k)3^
RM-M 400	7.26 ± 0.05	6.72 ± 0.07^b3,(i−k)3^	6.18 ± 0.07^a3,b3,(h–k)3^	5.52 ± 0.12^a3,b3,(h–k)3^	2.16 ± 1.32^a3^	3.24 ± 1.32^a3,b3,(h–k)3^	4.32 ± 1.08^a3^	5.40 ± 1.13^a3^
RM-H 8.75	7.26 ± 0.05	6.72 ± 0.07^b3,(i−k)3^	6.18 ± 0.07^a3,b3,(h–k)3^	5.52 ± 0.12^a3,b3,(h–k)3^	2.16 ± 1.32^a3^	3.24 ± 1.32^a3,b3,(h–k)3^	4.32 ± 1.08^a3^	4.50 ± 1.13^a3^
RM-H 17.50	7.26 ± 0.05	6.72 ± 0.07^b3,(i−k)3^	6.18 ± 0.07^a3,b3,(h–k)3^	5.52 ± 0.12^a3,b3,(h–k)3^	1.08 ± 1.08^a3^	3.16 ± 1.32^a3,b3,(h–k)3^	4.32 ± 1.08^a3^	4.50 ± 1.13^a3^
RM-H 35.00	7.26 ± 0.06	5.4 ± 0.00^b1,(i−k)3^	2.16 ± 1.32^a3,b3,(i−k)3^	1.08 ± 1.08^a3^	1.08 ± 1.08^a3^	2.24 ± 1.32^a3^	4.32 ± 1.08^a3^	4.50 ± 0.00^a3^
Anemonin 8.75	7.32 ± 0.07	2.16 ± 1.32^a3^	0.00 ± 0.00^a3^	0.00 ± 0.00^a3^	0.00 ± 0.00^a3^	0.00 ± 0.00^a3^	0.00 ± 0.00^a3^	0.00 ± 0.00^a3^
Anemonin 17.50	7.38 ± 0.07	2.16 ± 1.32^a3^	0.00 ± 0.00^a3^	0.00 ± 0.00^a3^	0.00 ± 0.00^a3^	0.00 ± 0.00^a3^	0.00 ± 0.0^a3^	0.00 ± 0.00^a3^
Anemonin 35.00	7.38 ± 0.07	1.08 ± 1.08^a3^	0.00 ± 0.00^a3^	0.00 ± 0.00^a3^	0.00 ± 0.00^a3^	0.00 ± 0.00^a3^	0.00 ± 0.00^a3^	0.00 ± 0.00^a3^

Data are expressed as mean ± SEM; n = 5; a: compared to 1% DMSO (dimethyl sulfoxide), b: compared to DA, c: compared to RM-M 100, d: compared to RM-M 200, e: compared to RM-M 400, f: compared to RM-H 8.75, g: compared to RM-H 17.50, h: compared to RM-H 35.00, i: compared to anemonin 8.75, j: compared to anemonin 17.50, k: compared to anemonin 35.00; 1: *p* < 0.05, 2: *p* < 0.01, 3: *p* < 0.001; 1% DMSO: vehicle, DA: diminazene aceturate, RM-M: 80% methanol extract of *R. multifidus*, RM-H: hydro-distilled extract of *Ranunculus multifidus*

**Fig. 2:**
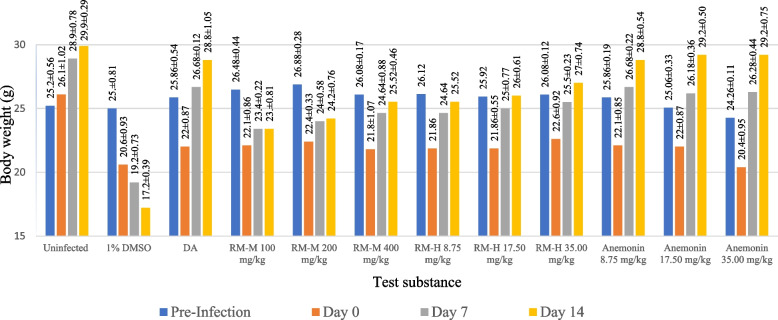
Body weight (g) of healthy uninfected and *Trypanosoma congolense*-infected mice treated with 80% methanol extract (RM-M), hydrodistilled extract (RM-H) of the leaves of *Ranunculus multifidus* and anemonin in in vivo antitrypanosomal assay. Data are expressed as mean ± SEM; n = 5; Pre-infection: just before infecting the mice; Day 0: just before starting treatment; Day 7: at the end of the treatment; and Day 14: at the end of the experiment; DMSO: dimethyl sulfoxide; DA: diminazene aceturate

Inhibition of growth of parasites caused by the test substances was supported by weight improvement of the experimental animals. In the present study, the experimental animals infected with *T. congolense* manifested weight loss. However, treatment with the crude extracts of *R. multifidus,* anemonin and diminazene aceturate prevented loss of body weight associated with parasitemia compared to negative controls. As shown in Fig. 2, the effect of anemonin was considerably superior to those of RM-M and RM-H. Weight gained by the positive control group and mice treated with anemonin was similar to body weight gain of the uninfected mice. The decreasing body weight in trypanosomiasis has been associated with decreased food intake, disturbed metabolic function and hypoglycemia [41]. An ideal antitrypanosomal agent would, therefore, prevent this occurrence. In the current study, anemonin showed weight maintenance effect.

**Fig. 3:**
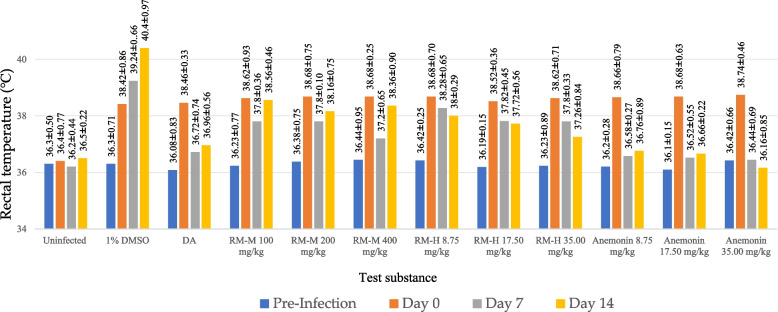
Rectal temperature (°C) of healthy uninfected and *Trypanosoma congolense*-infected mice treated with 80% methanol extract (RM-M), hydro-distilled extract (RM-H) of the leaves of *Ranunculus multifidus* and anemonin in in vivo antitrypanosomal assay. Data are expressed as mean ± SEM; n = 5. Pre-infection: just before infecting the mice; Day 0: just before starting treatment; Day 7: at the end of the treatment; and Day 14: at end of the experiment; DMSO: dimethyl sulfoxide; DA: diminazene aceturate

It is well documented that animals infected with trypanosomes exhibit fever. Further inference for the antitrypanosomal activity of the test substances was the lowering of rectal temperatures in the experimental animals. As depicted in Fig. 3, both extracts lowered rectal temperatures of the experimental animals compared to the negative control group (*p* < 0.001), although the effect of diminazene aceturate was superior (*p* < 0.001), with the exception of the 35.00 mg/kg concentration of RM-H, which showed comparable activity with that of the positive control. Interestingly, prevention of rectal temperature rise exerted by anemonin was markedly better (*p* < 0.001) than that of the positive control, diminazene aceturate. Increase in parasitemia level in animals usually results in decreased metabolic rates and develop severe hypothermia, which might result in death [41]. The results of the present study showed that anemonin possessed temperature stabilizing effect. In addition to parasite suppression this might indicate that anemonin controlled the immune system of infected mice along with controlling some pathological processes and balance the reduction in metabolic rate that produced drop in rectal temperature. This underscores its potential as a lead candidate for advancing the development of safer, more potent, and economically viable alternative treatment for trypanosomiasis.

**Fig. 4:**
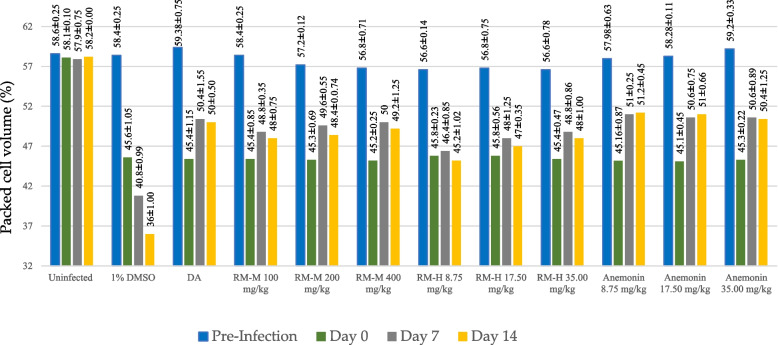
Packed cell volume (%) of healthy uninfected and *Trypanosoma congolense*-infected mice treated with 80% methanol extract (RM-M), hydro-distilled extract (RM-H) of the leaves of *Ranunculus multifidus* and anemonin in in vivo antitrypanosomal assay. Data are expressed as mean ± SEM; n = 5; Pre-infection: just before infecting the mice; Day 0: just before starting treatment; Day 7: at the end of the treatment; and Day 14: at end of the experiment; DMSO: dimethyl sulfoxide; DA: diminazene aceturate

As depicted in Fig. 4, *T. congolense* caused a significant packed cell volume (PCV) reduction in infected mice. The decrease in PCV value for untreated control is likely to be due to anemia which is a typical clinical manifestation of African trypanosomiasis [44]. Administration of the extracts to the infected mice produced a marked improvement in PCV when compared to the untreated uninfected mice. This has certainly protected the experimental animals from anemia, which is the underlying cause of death in infected animals [45]. However, neither the positive control nor the plant extracts and anemonin were able to reverse PCV of the infected mice to normal values.

Anemonin, a naturally occurring tri-spirocyclic dibutenolide, is commonly found in plants that belong to the *Ranunculaceae* family. It is a homodimer of protoanemonin, which is produced through the enzymatic breakdown of ranunculin when plant material is crushed. Anemonin has been shown to display robust chemosuppressive effects on malaria parasites and helps maintain key pathological indicators of malaria-infected mice [13].

The mechanism(s) by which the test substances exert their antitrypanosomal activity could be by interfering with the redox balance of the parasites acting on the cellular defences against oxidative stress. It is an established fact that trypanosomatids such as *Leishmania* and *Trypanosoma* utilize the vital enzyme γ-glutamylcysteine synthetase, which catalyzes the first and rate limiting step in the trypanothione biosynthic pathway, the ATP-dependent ligation of L-glutamate and L-cysteine to form γ-glutamylcysteine [46]. γ-Glutamylcysteine conjugate with L-glycine to form glutathione (GSH). GSH and the aliphatic polyamide spermidine conjugate to form trypanothione [47]. The trypanothione biosynthesis pathway is a unique metabolic pathway essential for survival of kinetoplastids such as trypanosomes [48]. Trypanothione plays a crucial role in regulation of intracellular thiol redox balance and in defense against chemical and oxidative stress [49]. Since trypanothione is absent in humans, trypanothione biosynthesis pathway is a potential drug target [47, 50, 51].

**Fig. 5:**
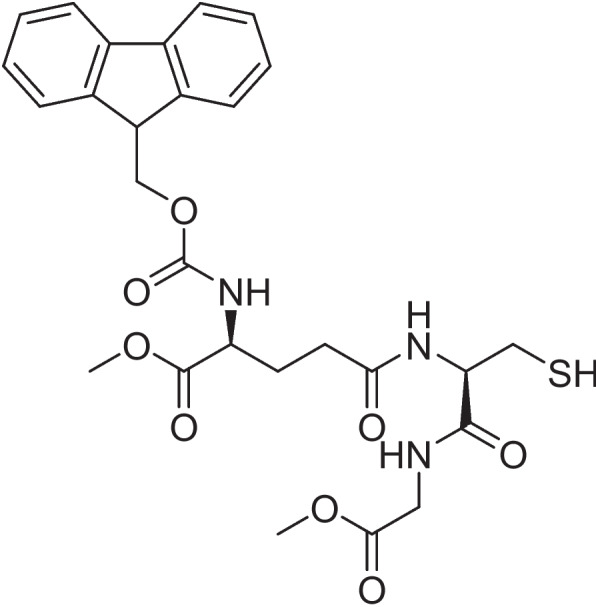
Fmoc-Protected glutathione methylester that was incubated with anemonin in DMSO solution

**Fig. 6:**
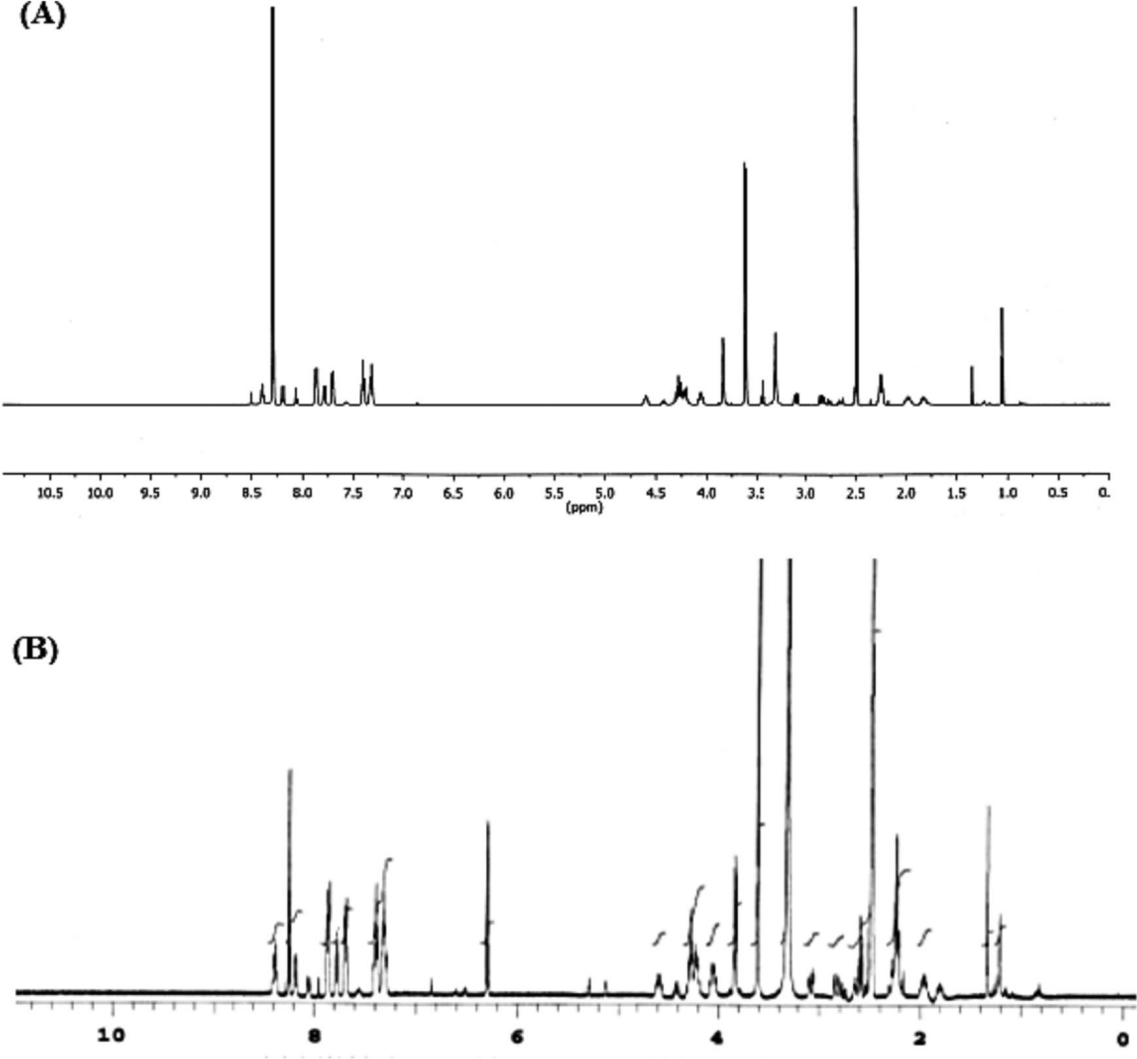
^1^H NMR spectra of (**A**) Fmoc-protected glutathione methylester (GT3) (**B**) Fmoc-protected glutathione methylester (GT3) after incubation with anemonin at 60 °C for 1 h

To test the hypothesis that the molecular mechamism of anemonin consists in reacting with thiol groups of parasitical metabolites and enzymes, anemonin was incubated with the Fmoc-protected dimethylester of glutathione (Fig. 5) in DMSO-d_6_, following the reaction by proton NMR. This glutathione derivative was used so that both reaction partners were soluble in the reaction medium. After seven days of incubation at room temperature, followed by 1 h at 60 °C, the proton NMR remained unchanged (Fig. 6A and B). It therefore seems unlikely that anemonin reacts with thiol groups without enzymatic catalysis. It remains to be investigated if anemonin reacts with other nucleophilic groups, such as the amino group of essential biomolecules. Its relative inertness towards thiol groups could explain why anemonin is not generally toxic, but only towards certain infective agents.

The main aim of the current study was to identify antitrypanosomal compound(s) from the leaf extract of *R. multifidus*, which is widely used as a traditional medicine to treat trypanosomiasis in south-west Ethiopia [10, 11]. Anemonin, identified in the plant as a major compound, demonstrated a significant trypanocidal activity eliminating parasites at a dose as low as 8.75 mg/kg/day and prevented relapse. This is interesting because at a dose of 28 mg/kg/day the standard drug diminazene aceturate also cleared the parasites but relapse occurred in all mice on day 12–14 post-treatment initiation. The present study also showed that anemonin does not react with thiol groups of parasitical metabolites and enzymes without enzymatic catalysis. However, further mechanistic studies are necessary to establish a complete profile of actions of anemonin as well as to confirm its toxicity and explore its beneficial scientific uses. Our current and previous findings demonstrated that anemonin is active against *T. congolense* and other protozoan parasites [13, 52], which therefore makes it a promising lead compound for new chemotherapies against infections caused by protozoan parasites.

## Conclusions

The present study confirmed that the fresh leaf extracts of *R. multifidus* are endowed with genuine in vitro and in vivo antitrypanosomal activity upholding the ethnobotanical use of the plant for the treatment of animal trypanosomiasis. Judging from the activity demonstrated by anemonin, it is possible to conclude that anemonin is responsible, in full or in part, for the trypanocidal activity of the plant. Furthermore, anemonin appears to have the potential to be used as a lead compound for the development of safe and cost-effective antitrypanosomal drug.

### Supplementary Information

**Additional file 1: Figure S1.** Atmospheric pressure chemical ionization mass spectrum of RM-H1 (Anemonin). **Figure S2.** Fourier-transform infrared spectrum of anemonin**. Figure S3.**^1^H-NMR spectrum of Anemonin. **Figure S4.**^13^C-NMR spectrum of Anemonin. **Figure S5.** DEPT-135 spectrum of anemonin**.**

## Data Availability

The data used to support the findings of this study are available from the corresponding author upon request.
