# Supplementary material for: In vitro and in vivo antitrypanosomal activity of the fresh leaves of Ranunculus Multifidus Forsk and its major compound anemonin against Trypanosoma congolense field isolate
Source: BMC Vet Res. 2024 Jan 27;20:32. doi: 10.1186/s12917-023-03856-1 (PMC10821574; doi:10.1186/s12917-023-03856-1)
Supplement: Supplementary file 1 — Additional file 1: Figure S1. Atmospheric pressure chemical ionization mass spectrum of RM-H1 (Anemonin). Figure S2. Fourier-transform infrared spectrum of anemonin. Figure S3.1H-NMR spectrum of Anemonin. Figure S4.13C-NMR spectrum of Anemonin. Figure S5. DEPT-135 spectrum of anemonin. [file 12917_2023_3856_MOESM1_ESM.docx]

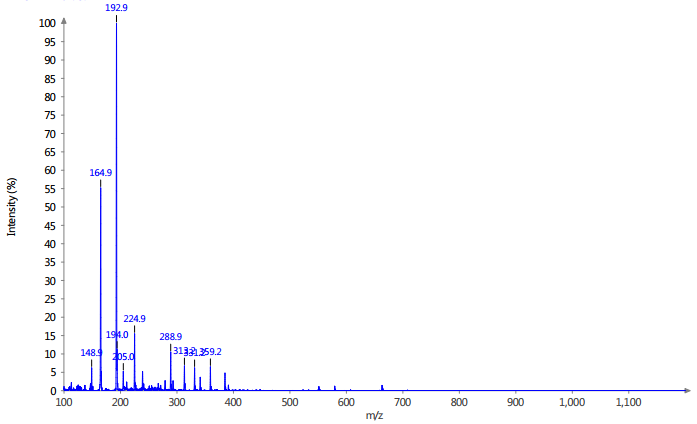


Figure S1. Atmospheric pressure chemical ionization mass spectrum of RM-H1 (Anemonin)

^
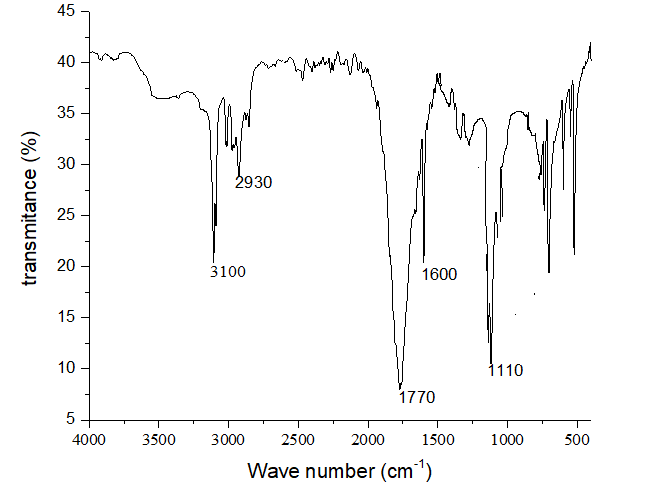
^

Figure S2. Fourier-transform infrared spectrum of anemonin


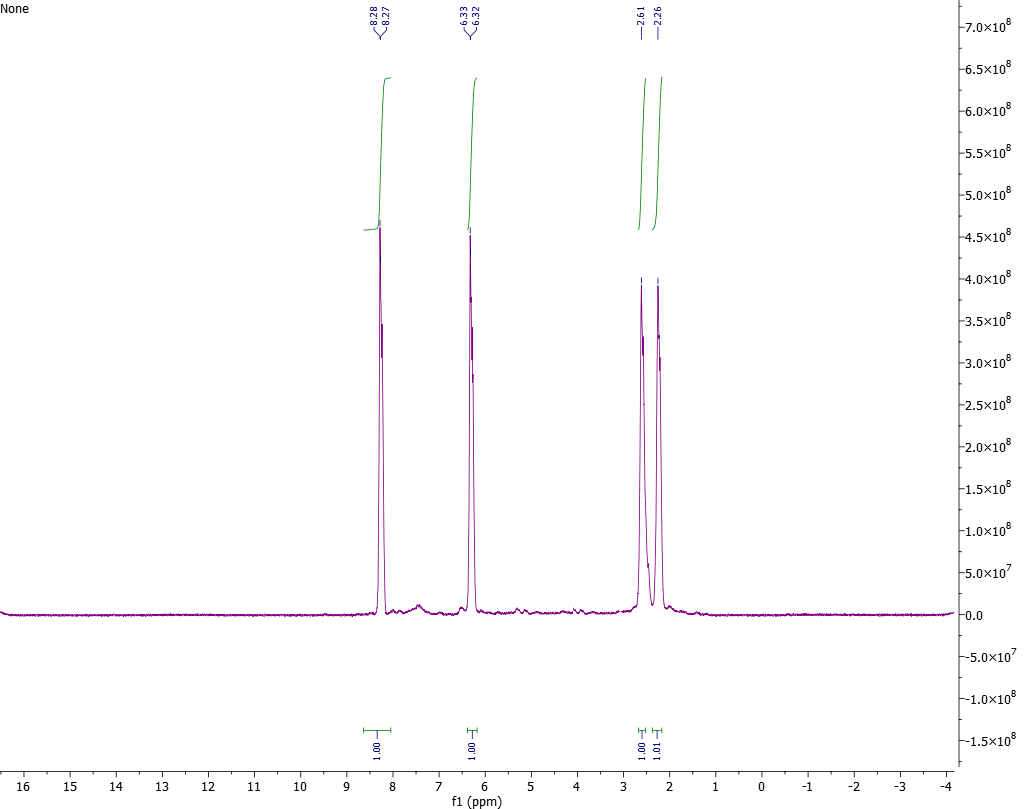


Figure S3. ^1^H-NMR spectrum of Anemonin

Figure S4. ^13^C-NMR spectrum of Anemonin

Figure S5. DEPT-135 spectrum of anemonin
